# Genomic upregulation of cardiac Cav1.2α and NCX1 by estrogen in women

**DOI:** 10.1186/s13293-017-0148-4

**Published:** 2017-08-14

**Authors:** Rita Papp, Glenna C. L. Bett, Agnieszka Lis, Randall L. Rasmusson, István Baczkó, András Varró, Guy Salama

**Affiliations:** 1 Department of Bioengineering and the Department of Medicine, Heart and Vascular Institute, University of Pittsburgh, Pittsburgh, PA 15261 USA; 2 Center for Cellular and Systems Electrophysiology, University at Buffalo, State University of New York, Buffalo, NY 14214 USA; 3 Department of Physiology and Biophysics, University at Buffalo, State University of New York, Buffalo, NY 14214 USA; 4 Obstetrics-Gynecology, University at Buffalo, State University of New York, Buffalo, NY 14214 USA; 5 Department of Pharmacology and Pharmacotherapy, University of Szeged, Szeged, Hungary; 6 MTA-SZTE Research Group for Cardiovascular Pharmacology, Hungarian Academy of Sciences, Szeged, Hungary; 7 Current Address: Ludwig Boltzmann Institute for Lung Vascular Research, Graz, Austria; 8 Department of Bioengineering and the Department of Medicine, Heart and Vascular Institute, University of Pittsburgh, 3550 Terrace Street, S628 Scaife Hall, Pittsburgh, PA 15261 USA

**Keywords:** Sex differences in the human heart, Sex differences in arrhythmia phenotype, 17-β-estradiol, L-type Ca^2+^ channel, Sodium-calcium exchanger, Regional heterogeneities in the heart

## Abstract

**Background:**

Women have a higher risk of lethal arrhythmias than men in long QT syndrome type 2 (LQTS2), but the mechanisms remain uncertain due to the limited availability of healthy control human tissue. We have previously reported that in female rabbits, estrogen increases arrhythmia risk in drug-induced LQTS2 by upregulating L-type Ca^2+^ (I_Ca,L_) and sodium-calcium exchange (I_NCX_) currents at the base of the epicardium by a genomic mechanism. This study investigates if the effects of estrogen on rabbit I_Ca,L_ and I_NCX_ apply to human hearts.

**Methods:**

Postmortem human left ventricular tissue samples were probed with selective antibodies for regional heterogeneities of ion channel protein expression and compared to rabbit myocardium. Functionally, I_Ca,L_ and I_NCX_ were measured from female and male cardiomyocytes derived from human induced pluripotent stem cells (iPS-CMs) with the voltage-clamp technique from control and estrogen-treated iPS-CMs.

**Results:**

In women (*n* = 12), Cav1.2α (primary subunit of the L-type calcium channel protein 1) and NCX1 (sodium-calcium exchange protein) levels were higher at the base than apex of the epicardium (40 ± 14 and 81 ± 30%, respectively, *P* < 0.05), but not in men (*n* = 6) or postmenopausal women (*n* = 6). Similarly, in cardiomyocytes derived from female human iPS-CMs, estrogen (1 nM, 1–2 days) increased I_Ca,L_ (31%, *P* < 0.05) and I_NCX_ (7.5-fold, − 90 mV, *P* < 0.01) and their mRNA levels (*P* < 0.05). Moreover, in male human iPS-CMs, estrogen failed to alter I_Ca,L_ and I_NCX_.

**Conclusions:**

The results show that estrogen upregulates cardiac I_Ca,L_ and I_NCX_ in women through genomic mechanisms that account for sex differences in Ca^2+^ handling and spatial heterogeneities of repolarization due to base-apex heterogeneities of Cav1.2α and NCX1. By analogy with rabbit studies, these effects account for human sex-difference in arrhythmia risk.

## Background

Sex-dependent arrhythmia risks are particularly pronounced when there is repolarization delay such as in bradycardia and congenital or drug-induced long QT syndrome type 2 (LQTS2). Prolongation of the cardiac action potential (AP) is caused by an imbalance between the depolarizing and repolarizing ionic currents resulting in an accompanying prolongation of the QT interval on the EKG. Congenital long QT type 2 (LQT2) is caused by mutations of the Kv11.1 K^+^ channel protein (hERG). This results in a loss of function of the rapid component of the delayed rectifying K^+^ current, I_Kr_, thus prolonging AP duration (APD) and QT interval [1]. Although the incidence of all forms of congenital LQT is rare (<1/5000), drug-induced LQTS remains a particularly serious public health problem in terms of safety pharmacology [2–4] because a wide range of cardiac and non-cardiac drugs suppress I_Kr_, prolong APDs, and promote early afterdepolarizations (EADs) that lead to potentially lethal torsade de pointes (TdP) [1, 5–8]. Women are at much greater risk than men of experiencing drug-induced LQTS and developing TdP [9–11].

There is general agreement that torsade de pointes is initiated by EADs. “Early” EADs that occur early during the plateau phase of the AP may be caused by the spontaneous reactivation of L-type Ca^2+^ channels [12], whereas “late” EADs that arise in a later phase of the AP plateau are caused by an imbalance between the Ca^2+^ influx and efflux resulting in sarcoplasmic reticulum (SR) Ca^2+^ overload, spontaneous SR Ca^2+^ release, and activation of the forward mode of the Na-Ca exchanger (NCX) current which can further prolong the AP and trigger EADs by the reactivation of the L-type Ca^2+^ current (I_Ca,L_) [13, 14].

A salient feature of LQT2 is the marked sex difference in arrhythmia risk: women are twice as likely as men to experience TdP as a result of inherited LQT2 or drug-induced LQT [11, 15, 16]. These differences disappear in postmenopausal women over the age of 50 [9, 15, 16]. It should be noted that postmenopausal women with congenital LQT2 have a higher recurrence of syncope, and these repeated syncope events were not attributed to TdP [17]. Sex differences in arrhythmia risk have been attributed to a reduced “repolarization reserve” in adult women, consistent with their longer rate-corrected QT (QTc) intervals and hence greater susceptibility to I_Kr_ blockade than men [10, 18]. However, the QT interval is not an accurate predictor of arrhythmia risk, with asymptomatic individuals having long QT and conversely individuals with QT intervals in the normal range exhibiting severe arrhythmias [19]. A major challenge to unraveling the molecular mechanisms underlying LQT-related arrhythmias is the lack of available human myocardial tissues. As a result, animal models have been extensively used to study human sex differences, but data are difficult to interpret due to differences in ion channels expressed in the heart, different effects of sex steroids, and different propensities to arrhythmia. In a recent analysis of the data, we proposed that the rabbit may be the best surrogate to study sex differences in humans [20].

The higher risk of LQT2-related arrhythmias in women is likewise observed in female New Zealand White rabbits compared to their male counterparts [21], and the risk is reversed in both pre-pubertal rabbits [22] and man [23]. In female rabbits, ovariectomy was protective of drug-induced LQT2-related arrhythmias whereas 17-β-estradiol replacement reversed the protective effects of ovariectomy and promoted EADs and arrhythmias [24–26]. These studies suggest that 17-β-estradiol promotes TdP in female hearts.

Dual optical mapping of action potentials (APs) and intracellular free Ca^2+^ (Ca_i_) transients in a Langendorff rabbit model of drug-induced LQT2 revealed that adult female hearts were more prone to EADs and TdP and that the origins of EADs and Ca_i_ oscillations preceded the occurrence EADs [27, 28] [21]. We also showed that in freshly isolated ventricular myocytes, peak I_Ca,L_ density and Cav1.2α (primary subunit of the L-type Ca^2+^ channel protein) channel protein were 25–30% greater at the base than the apex of adult female compared to male hearts [21]. Western blot analysis and voltage-clamp studies showed that the higher level of Cav1.2α at the base of the adult female heart was associated with a regional elevation of NCX and I_NCX_ [14, 21, 29]. These findings are congruent with data from humans since cardiac contractility is greater in women than men [30] and myocytes isolated from women post-transplantation have a tendency to have greater I_Ca,L_ than men [31].

Moreover, incubation of myocytes with 17-β-estradiol (E2; 1 nM) revealed a regional genomic upregulation of NCX and Cav1.2α, mRNA, protein levels, and their respective current densities, I_NCX_ and I_Ca,L_. These effects are mediated by estrogen receptors, enhanced transcription and protein biosynthesis of NCX, and Cav1.2α channel protein [14, 32]. We extended these studies by showing that E2 upregulates I_Ca,L_ in cultured myocytes isolated from the base of female hearts by a genomic mechanism mediated via the α isoform of estrogen receptors (ERα) but not via the β isoform (ERβ) [32].

Our findings in rabbit hearts [21] were consistent with a report of endocardium-to-epicardium dispersion of I_Ca,L_ in female but not in male rabbit hearts and with I_Ca,L_ density being higher on the epicardium than the endocardium and no male-female differences on the endocardium [33].

These findings raise the question of whether in men similar mechanisms explain sex differences in arrhythmia risk in LQTS. Here, we analyzed heterogeneities in the expression of ion channels and Ca^2+^-transport proteins from the base and apex of the left ventricular epicardium of healthy human donor hearts. In addition, the possible role of estrogen-mediated genomic regulation of protein expression was evaluated (a) indirectly by introducing a third study group of postmenopausal women who were older than 50 years and thus expected to have significantly lower levels of estrogen than younger adult women and (b) treating female and male human cardiac myocytes derived from induced pluripotent stem cells with E2 followed by voltage-clamp measurements of I_Ca,L_ and I_NCX_ and their corresponding mRNA levels.

## Methods

### Human and rabbit tissue samples

Hearts were obtained from organ donors whose non-diseased hearts were explanted to obtain pulmonary and aortic valves for transplant surgery. Before cardiac explantation, organ donors did not receive medication apart from dobutamine, furosemide, and plasma expanders.

The investigations conformed to the principles of the Declaration of Helsinki. The experimental protocols were approved by the University of Szeged and National Scientific and Research Ethical Review Boards (No. 51-57/1997 OEj) and by the Scientific and Research Ethical Committee of the Medical Scientific Board at the Hungarian Ministry of Health (ETT-TUKEB) under ethical approval No. 4991-0/2010-1018EKU (339/PI/010).

After explantation, each heart was perfused with cardioplegic solution and kept cold (4–6 °C) for 2–4 h prior to dissection. Tissue samples were excised from the subepicardial and subendocardial layers of the left ventricular base and the subepicardium of the left ventricular apex. Samples were flash-frozen in liquid nitrogen and stored at − 80 °C until use. Three groups of myocardial samples were analyzed: adult men (*n* = 6), adult women (between the ages of 17 and 49 years, *n* = 11), and postmenopausal women (>50 years, *n* = 6); the minimum, maximum, and mean ages for each group are shown by Table 1.

**Table 1:** Summary of the number of individuals (*n*) and ages of the human used in the study

Group	Men (*n* = 6)	Adult women (*n* = 11)	Postmenopausal women (*n* = 6)
Mean age (years)	45	34	56
Min-Max (years)	32–58	17–49	51–68

For rabbit cardiac samples, 3-month-old male (*n* = 2) and female (*n* = 3) New Zealand White rabbits were heparinized (200 U/kg i.v.) and euthanized with an intravenous injection of sodium pentobarbital (50 mg/kg). The hearts were excised and perfused through the aorta with ice-cold Tyrode’s solution for 5 min, then cardiac tissue samples were harvested from the base and apex of the left ventricular epicardium, as previously described [21]. In previous reports, the base-apex heterogeneities of Cav1.2α1 and NCX1 (sodium-calcium exchange protein) on the epicardium were shown to occur in female but not male adult rabbit hearts [21]. We also reported spatial heterogeneities along the right ventricle and included the spatial heterogeneities of SERCA2a, RyR2, Nav1.5, and hERG [29]. Here, we probed all the above channels (see Fig. 2) from the left ventricles of a new set of male and female adult rabbits.

Protocols were approved by the University of Pittsburgh Institutional Animal Care and Use Committee and were in accordance with the current Guide for the Care and Use of Laboratory Animals published by the National Institutes of Health. These studies also comply with the procurement of the rabbits, and the animal husbandry and the experiments conform to the “European Convention for the Protection of Vertebrate Animals used for Experimental and other Scientific Purposes” (Council of Europe No 123, Strasbourg 1985). Western blot results were pooled together with earlier Cav1.2α1 [21, 32] and NCX1 data [14], while, for the other proteins, earlier samples from 3 female and 3 male hearts were run in addition to our freshly isolated samples, reaching a total n number of *n* = 6 for female and *n* = 5 for male rabbits.

### Western blots

Cardiac tissue samples were pulverized in liquid nitrogen; then approximately 100 mg of tissue powder were suspended in lysis buffer containing (in mM) 150 NaCl, 1 EDTA, 2.5 MgCl_2_, 20 HEPES, 1% Triton X-100, and 0.1% SDS as well as protease inhibitor cocktail and PMSF (both from Sigma), then homogenized with a glass-teflon homogenizer. After 20-min centrifugation at 16,000*g*, the supernatants were collected, and the protein concentration was determined with the Bradford protein assay (Bio-Rad Laboratories, Hercules, CA, USA). Fifty micrograms of total protein samples were ran on 4–15% polyacrylamide gels (Bio-Rad), then transferred to PVDF membranes (Millipore). After blocking with 5% milk, the membranes were incubated overnight with primary antibodies against Cav1.2α1 (rabbit: Santa Cruz sc103588, 1:100; human: custom antibody from Zymed, 1:250), NCX1 (Thermo Scientific MA3-926, 1:500), SERCA2a (Santa Cruz sc53010, 1:1000), RyR2 (Thermo Scientific MA3-916, 1:500), Nav 1.5 (Alomone Laboratories ASC-005, 1:100), hERG (Santa Cruz sc15968, 1:100), and GAPDH (glyceraldehyde 3-phosphate dehydrogenase; Abcam ab70136, 1:1000). After incubation with alkaline phosphatase conjugated secondary antibodies (Jackson ImmunoResearch), blots were developed with the Lumi-Phos reagent (Thermo Scientific), then imaged and analyzed with the Image Lab GelDoc system (Bio-Rad). Band intensities were normalized for GAPDH as a loading control.

In order to immunolabel Cav1.2α1 in human samples, several antibodies were tested including SC 103588 from Santa Cruz, ACC-003 from Alomone, and Ab 81980 and Ab 84814 from Abcam; however, these antibodies did not give satisfactory results. Therefore, a custom-made antibody produced by Zymed was used, which gave a single band around 250 kDa, and this band disappeared when the antibody was pre-incubated with the immunogen peptide.

Protein expression was compared for the three groups (4 men, 6 adult females, and 4 postmenopausal females) between the base and apex of the left ventricular epicardium, the epi, and the endocardium of the left ventricular base. Left ventricular samples from the base of the three groups were run on the same gel.

### iPS-CM cell culture

Commercially available human male and female iPSC-derived cardiac myocytes (iPS-CMs, Axol Bioscience, UK) were prepared according to the manufacturer’s instructions. Briefly, cardiac myocytes (1 ml, stored frozen in liquid N_2_) thawed in a 37 °C water bath were initially suspended in 1 ml Axol Complete Cardiomyocyte Medium (warmed to 37 °C). An additional 8 ml of medium was added and mixed by gentle inversion. Cardiac myocytes were seeded (25,000 cells/well) in 1.5 ml medium in 12 well culture plates that contained glass coverslips (15-mm diameter, Warner Instruments, CT) pre-coated with Matrigel™ (Corning Life Sciences, MA). Cells were incubated for 24 h at 37 °C, 7% CO_2_. Non-adherent cells were removed by rinsing with media. After 7 days in culture, 17-β-estradiol (Sigma) was added to the media at a final concentration of 1 nM. The iPS-CMs were kept in 17-β-estradiol for 24–48 h before measuring I_Ca,L_ or I_NCX_ by voltage-clamp technique. Control cells were treated with media containing DMSO (< 0.1%). Cultured iPS-CMs had a tendency to mature and exhibit spontaneous contractions by the 7th day of incubation. The male and female iPS-CMs used in this study had common features found in all iPS-CMs: spontaneous automaticity and contractions and stable ionic currents for weeks, as previously reported [34].

### Electrophysiology

Electrophysiological measurements were performed in the whole-cell voltage-clamp configuration. Glass recording electrodes with resistances of 1–1.5 MΩ were pulled from borosilicate glass (T1W150-4. World Precision Instruments, FL) using a Sutter micropipette puller. For recording I_Ca,L_, pipettes were filled with (in mM): 115 CsCl, 20 TEA-Cl, 1 MgSO_4_, 5 EGTA, 5 MgATP, 5 Tris-creatine PO_4_, 0.3 Tris-GTP, and 10 HEPES (pH 7.2). The external solution contained (in mM): 144 NMDG-Cl, 12 CsCl, 2 CaCl_2_, 1 MgCl_2_, 10 HEPES, and 10 glucose (pH 7.35). Cells were clamped at − 80 mV with currents recorded over a 500-ms voltage step from the holding potential to potentials between − 110 and 70 mV in increments of 10 mV. For recording I_NCX_, pipettes were filled with (in mM): 120 glutamic acid, 120 CsOH, 0.5 MgSO_4_, 20 NaCl, 10 EGTA, 3 CaCl_2_, 5 MgATP, and 10 HEPES (pH 7.2). The external solution contained (in mM): 140 NaCl, 10 CsCl_2_, 1 CaCl_2_, 1 MgCl_2_, 10 HEPES, and 10 glucose (pH 7.4). Nifedipine (10 μM) and ouabain (10 μM) were added to the external solution to inhibit I_ca,L_ and the Na-K ATPase, respectively. Cells were clamped at − 50 mV and then stepped to + 100 mV for 80 ms followed by a descending voltage ramp from + 100 to − 120 mV over 1600 ms. I_NCX_ was determined by measuring the Ni^2+^-sensitive current in the voltage ramp. The residual currents measured in the presence of 5 mM Ni^2+^ were subtracted from the total current recorded in the absence of Ni^2+^. All measured currents were normalized to the cell capacitance.

### qRT-PCR

On days 2, 3, and 4 after initial treatment with beta-estradiol, RNA was isolated from treated and vehicle (DMSO) control wells using a RNeasy Micro Kit (Qiagen) according to the manufacturer’s instruction. cDNA was prepared from the isolated RNA (1 μg) using a QuantiTect Reverse Transcription kit (Qiagen). Table 2 shows the nucleotide sequence of the primers used in the study. Quantitative real-time PCR (qPCR) was performed using the iQ SYBR Green Supermix (Bio-Rad). Samples were amplified in triplicate. Amplifications were performed using the MyIQ Real-time PCR detection system (Bio-Rad). Conditions were as follows: 50 °C for 2 min, 1 cycle; 95 °C for 3 min, 1 cycle; 95 °C for 15 s ➔ 60 °C for 1 min, 40 cycles; followed by a melting curve analysis: 55–95 °C in 0.5 °C increments for 2 s a step. Data was analyzed using IQ 5 Optical System Software (Bio-Rad), and Excel using the ΔΔC_t_ method.

**Table 2:** Nucleotide sequence of primers used

GAPDH-F	5'-AAT TGA GCC CGC AGC CTC CC-3'
GAPDH-R	5'-GAG CGA TGT GGC TCG GCT GG-3'
Troponin-F	5'-CAA GCA GGT GAA GAA GGA GG-3'
Troponin-R	5'-CAG TAG GCA GGA AGG CTC AG-3'
Cav1.2α-R	5'-GCC TAC CTC CGC AAC GGC TG-3'
Cav1.2α-F	5'-CGG CCC CTT TCC CTC CGA GA-3'
NCX-F	5'-ACC TGT TTG GCC AAC CTG TCT TCA-3'
NCX-R	5'-TGC TGG TCA GTG GCT GCT TGT-3

### Statistical analysis

Data are shown as mean ± SEM of the percentage differences between the base and apex or between the epicardium and endocardium, respectively. Base vs apex and epicardial vs endocardial protein levels were compared with the paired *t* test, and differences were considered significant at *P* ≤ 0.05. Patch clamp data are shown as mean ± SEM normalized to cell capacitance. Results were compared with the unpaired *t* test and were considered significant at *P* ≤ 0.05.

## Results

**Fig. 1:**
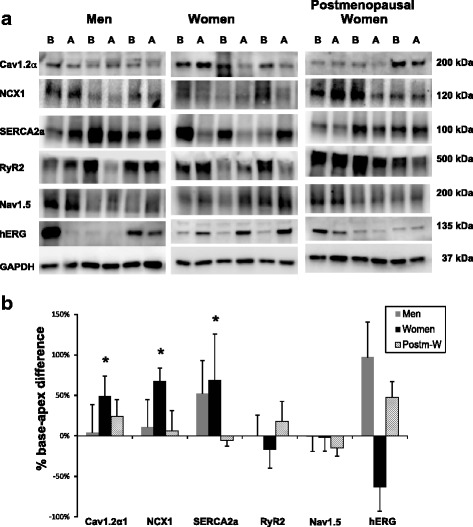
Comparison of protein expression between the base (*B*) and apex (*A*) of the left ventricular epicardium in humans. Protein samples from the base (*B*) and the apex (*A*) of the LV epicardium were obtained from 3 men, 3 women, and 3 postmenopausal women and were probed with antibodies to compare the relative expression of Cav1.2α, NCX1, SERCA2a, RyR2, Nav1.5, and hERG. Panel **a** shows the protein densities which were normalized with respect to GAPDH. Panel **b** is a histogram of normalized band intensities expressed as base-apex percent differences derived from an analysis of myocardium from 6 men, 11 women, and 6 postmenopausal women

**Fig. 2:**
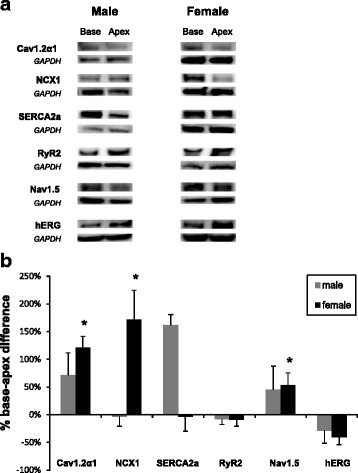
**a** Western blots of base-apex differences in channel protein expression in male and female rabbit left ventricular epicardium. **b** Base-apex percent differences in protein expression on the rabbit left ventricular epicardium

From left ventricular epicardial tissue samples, we found that adult women (*n* = 11), but not men (*n* = 6) or postmenopausal women (*n* = 6), had a higher expression of Cav1.2α1 and NCX1 (40 ± 14 and 81 ± 30%, respectively, *P* < 0.05) at the base compared to the apex (Fig. 1a, b). These regional differences were similar to those found in female rabbits (106 ± 50 and 185 ± 53%, respectively) (Fig. 2). In women, SERCA2a was also more abundant at the base compared to the apex (130 ± 67%), and this large spatial gradient was not observed in men or postmenopausal women. In contrast, female rabbits did not have a difference in SERCA2 expression, but male rabbit hearts had significantly higher expression of SERCA2a at the base than apex (Fig. 2). On the other hand, Nav1.5 protein expression, which exhibited a base-apex difference in female rabbits, was homogeneous throughout the epicardium in all human groups. No significant differences in the expression of RyR2 occurred in any group, in any species. In rabbits, the expression of rERG was higher at the apex in both sexes. In contrast, hERG expression in humans had a similar tendency (statistically not significant) in women (Fig. 1), whereas, in men and postmenopausal women, hERG had a tendency to be more abundant at the base. Table 3 summarizes the distribution of key ion channel proteins in human myocardium and calculations regarding their base-apex differences.

**Table 3:** Distribution of key ion channel proteins in human myocardium and calculations regarding base-apex differences

Protein	Group	Base	Apex	% Difference	*P* value
Cav1.2α	Men	0.49 ± 0.1	0.47 ± 0.1	4.1%	1.0000
	Adult women	0.57 ± 0.12	0.38 ± 0.09	48.8%*	0.0002
	Postm. women	0.74 ± 0.25	0.59 ± 0.17	24.1%	0.5625
NCX1	Men	0.71 ± 0.07	0.64 ± 0.13	10.9%	0.6875
	Adult women	0.68 ± 0.18	0.41 ± 0.12	67.4%*	0.0024
	Postm. women	0.67 ± 0.1	0.63 ± 0.15	6%	0.8438
SERCA2a	Men	2.67 ± 1.19	1.76 ± 0.37	52.2%	1.0000
	Adult women	2.43 ± 0.59	1.44 ± 0.33	68.7%*	0.0398
	Postm. women	0.94 ± 0.23	0.99 ± 0.28	−5.8%	0.5625
RyR2	Men	0.95 ± 0.28	0.94 ± 0.14	0.5%	1.0000
	Adult women	0.41 ± 0.11	0.5 ± 0.11	−17.3%	0.3804
	Postm. women	0.26 ± 0.09	0.22 ± 0.04	17.6%	0.6875
Nav1.5	Men	1.96 ± 0.35	1.97 ± 0.33	−0.8%	1.0000
	Adult women	1.16 ± 0.2	1.18 ± 0.28	−1.8%	0.8394
	Postm. women	1.03 ± 0.13	1.21 ± 0.19	−15%	0.2188
hERG	Men	0.19 ± 0.09	0.09 ± 0.03	97%	0.6250
	Adult women	0.17 ± 0.08	0.46 ± 0.24	−63.4%	0.1230
	Postm. women	0.35 ± 0.15	0.24 ± 0.09	47.8%	0.1250

Group mean ± SEM density values for each protein, normalized with GAPDH, and % base-apex differences in the human left ventricular epicardium. * *P* < 0.05, Wilcoxon matched-pairs signed rank test.

**Fig. 3:**
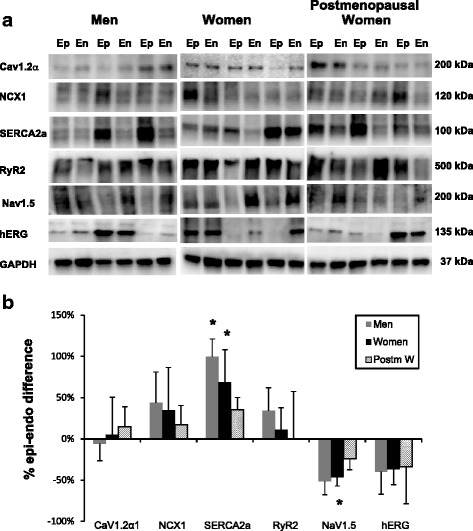
Comparison of protein expression between the epicardium (*Ep*) and endocardium (*En*) of the left ventricular myocardium in human hearts. Protein samples from the epicardium (*Ep*) and endocardium (*En*) of the LV were obtained from 3 men, 3 women, and 3 postmenopausal women and were probed with antibodies to compare the relative expression of Cav1.2α, NCX1, SERCA2a, RyR2, Nav1.5, and hERG. Panel **a** shows the protein densities which were normalized with respect to GAPDH. Panel **b** is a histogram of normalized band intensities expressed as epi-endo percent differences derived from an analysis of myocardium from 6 men, 11 women, and 6 postmenopausal women

Cav1.2α and NCX1 showed a tendency to be more abundant on the epicardium compared to the endocardium, but these differences did not reach statistical significance in any of the groups (Fig. 3). SERCA2a was significantly higher on the epicardium compared to the endocardium, but this effect was not sex-related since it occurred in all the three human study groups (men 197 ± 69%, adult women 131 ± 39%, postmenopausal women 190 ± 105%). Nav1.5 showed a tendency to be more abundant on the endocardium, but did not reach statistical significance except in postmenopausal women. hERG had a tendency of higher levels on the endocardium that did not reach statistical significance.

### Effects of E2 on human iPS-CMs

**Fig. 4:**
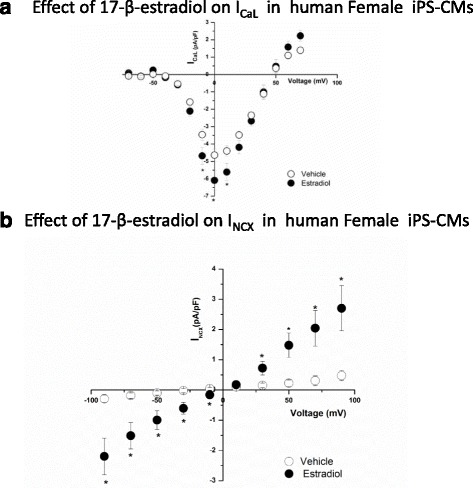
Effect of E2 incubation on I_Ca,L_ and I_NCX_ of female human iPS-CMs. **a** I_Ca,L_ current-voltage relationship from cardiac myocytes derived from hiPS cells was measured in cells cultured with 1 nM E2 (*filled circles*, *n* = 13) and compared to those cultured with vehicle (*empty circles*, *n* = 15). I_Ca,L_ was significantly increased by E2 incubation. (*P < 0.05). **b** I_NCX_ was measured as a function of voltage in female human iPS-CMs that were incubated with estradiol (*filled circles*, *n* = 5) and compared to iPS-CMs incubated with the vehicle (control) (*empty circles*, *n* = 5). There was a significant increase in I_NCX_ when incubated with E2. *P < 0.05

Female cardiomyocytes derived from induced pluripotent stem cells (iPS-CM) were cultured with 1 nM 17-β-estradiol (E2) or vehicle (control) for 1–3 days; then I_Ca,L_ density was recorded as a function of membrane potential (V_m_). E2-treated myocytes had a significantly higher peak current compared to vehicle-treated iPS-CMs (from − 4.64 ± 0.35 (*n* = 15) to − 6.09 ± 0.55 pA/pF; *n* = 13, at 0 mV, *P* < 0.05) (Fig. 4a). Similarly, the Ni^2+^-sensitive Na-Ca exchange current, I_NCX_, was significantly larger in iPS-CMs treated with E2 (1 nM) compared to myocytes treated with vehicle (Fig. 4b. At − 90 mV, I_NCX_ current density in iPS-CMs incubated with E2 was − 2.20 ± 0.54 pA/pF (*n* = 5) compared to − 0.29 ± 0.13 pA/pF (*n* = 5, *P* < 0.01). The increases in I_Ca,L_ and I_NCX_ densities were accompanied with higher levels of mRNA for Cav1.2α (2.93 ± 0.57-fold increase, *n* = 10, *P* < 0.05) and for NCX1 (3.24 ± 1.22-fold increase, *n* = 9, *P* < 0.05) relative to glyceraldehyde 3-phosphate dehydrogenase (GAPDH), as a housekeeping gene.

**Fig. 5:**
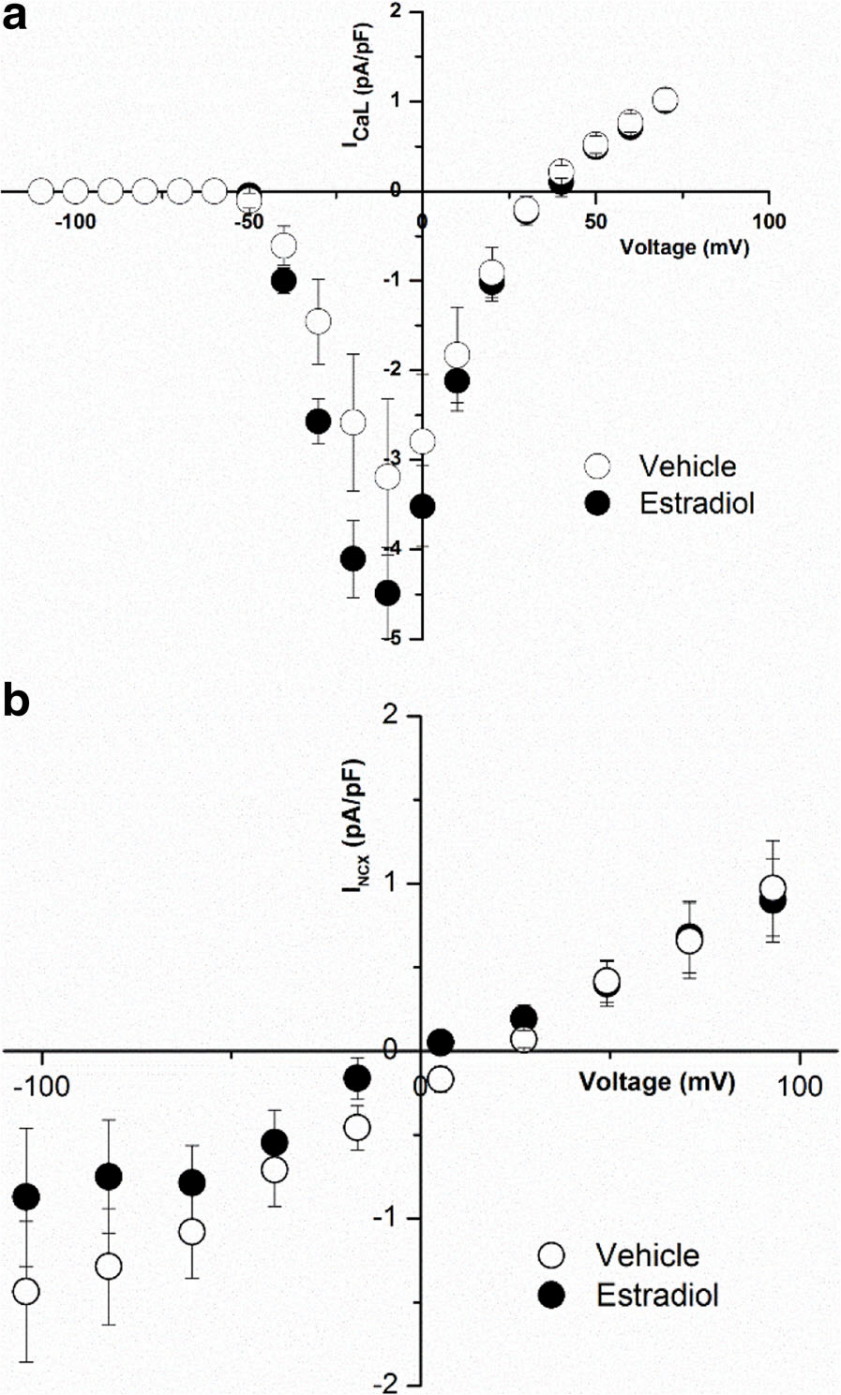
Effect of E2 incubation on I_Ca,L_ and I_NCX_ of male human iPS-CMs. **a** Effect of 17-β-estradiol on I_Ca,L_ in male iPS-CMs. I_Ca,L_ was measured as described in the methods in male iPS-CMs following treatment with 17-β-estradiol (*filled circles*) and vehicle (*empty circles*). 17-β-estradiol treatment displayed a small but not significant increase in I_Ca,L_ as compared to vehicle treatment. **b** Effect of 17-β-estradiol on I_NCX_ in male iPS-CMs. Shown are the averaged current density for I_NCX_ obtained from male iPS-CMs treated with 17-β-estradiol (*filled circles*) and vehicle (*empty circles*)

Male human iPS-CMs treated with 17-β-estradiol showed a trend for increased peak current density at − 10 mV as compared to vehicle-treated iPS-CMs (17-β-estradiol-treated − 4.49 ± 0.50 pA/pF, *n* = 5 vs. vehicle-treated − 3.19 ± 0.88, *n* = 5, NS at *P* < 0.05) (Fig. 5a). There was no effect of 17-β-estradiol on I_NCX_ in male myocytes as compared to vehicle (Fig. 5b).

## Discussion

Our earlier findings in female rabbits linked the initiation of EADs and TdP to regions at the base of the ventricles where there was higher expression of Cav1.2α1 and NCX1, their mRNA and their respective currents I_Ca,L_ and I_NCX_. Here, we analyzed the density of ion channel proteins in left ventricles from healthy human donor hearts and found that adult women, similar to female rabbits, have significantly higher levels of Cav1.2α1 and NCX1 at the base of the epicardium compared to the apex. This difference was sex- and age-dependent as it did not occur in adult men or in postmenopausal women supporting the interpretation that estrogen regulates the expression of these proteins. Moreover, female human cardiomyocytes derived from induced pluripotent stem cells responded to estrogen (1 nM) in the culture medium by a genomic upregulation (in 1–2 days) of mRNA levels and current densities of I_Ca,L_ and I_NCX_.

In female rabbit hearts, we recently showed that the higher expression of Cav1.2α and NCX1 produce aberrant systolic Ca^2+^ handling that trigger EADs and arrhythmias under conditions of repolarization delay, namely bradycardia [29] and drug-induced long QT [35]. Although the link between repolarization delay, Ca^2+^ overload, and arrhythmias was demonstrated in rabbit hearts, the same mechanism most likely applies to human hearts because sex differences in arrhythmia risk in rabbits closely match the profile of human arrhythmias associated with repolarization delays [20]. The base-apex heterogeneities of cellular Ca^2+^ handling are a result of an upregulation of I_Ca,L_ and I_NCX_ by 17-β-estradiol at myocytes isolated from the base of the ventricles [14, 21, 32]. The higher density of I_Ca,L_ and I_NCX_ is mediated by estrogen receptors (α and β isoforms: ERα and ERβ), requires 24–48 h, and was inhibited by blockers of transcription or translation; hence, estrogen acts via a genomic mechanism [14, 32]. Agonists of both ERα and ERβ upregulate NCX1, whereas only agonists of ERα upregulate Cav1.2α. Moreover, in silico analysis of the promoter region of human and rabbit CACNA1C detected 8 high-probability estrogen receptor ERα (but not ERβ) binding sites within the first 500 nucleotides of the promoter region (at http://genome.ucsc.edu and BIOBASE Knowledge Library). Chip-on-chip analysis of the human CACNA1C detected the same 8 high-quality ERα binding sites in the promoter region in human MCF-7 cells [36]. The analysis suggests that estrogen regulates the transcription of the gene that encodes for Cav1.2α in human and rabbit hearts via ERα (but not ERβ) by a “classical” genomic mechanism. Consistent with bioinformatics findings, we now show that estrogen upregulates I_Ca,L_ in isolated human iPS-derived cardiomyocytes.

An interesting unresolved question is why myocytes from the base respond to estrogen treatment but myocytes from the apex do not. A possible mechanism could be differences in the expression of receptor ERα and ERβ between the apex and base. However, we showed by Western blot analysis that both receptors are equally expressed at the base and apex of ventricles in both male and female myocardium [32]. A promising alternative mechanism to explain base-apex differences in the response to estrogen is to analyze base-apex differences in the activation of transcription factors from the same hearts, an approach that is only applicable to animal models.

Besides base-apex differences on the epicardium, we also examined epicardium-to-endocardium differences. In rabbit hearts, endocardial cells from the base did not respond to estrogen [14] and both Cav1.2α and NCX1 showed a tendency to be higher on the epicardium than the endocardium confirming an earlier study of higher expression of Cav1.2α on the female rabbit epicardium [37]. Transmural dispersion of repolarization measured with plunge electrodes was greater in female than male rabbits and contributed to the higher incidence of torsade de pointes in female hearts [38].

In dog hearts, I_NCX_ and I_Ca,L_ densities were greater on the epicardium than endocardium [39] and the sarcoplasmic reticulum (SR) Ca^2+^ load was higher in the epicardium. The higher I_NCX_ and SR load is consistent with the higher SERCA2a levels on the epicardium of human hearts, which was found in non-failing and failing hearts [40] and in normal male and female hearts (Fig. 3). Nav1.5 was found to be higher on the endocardium by Gaborit et al. [41], and we show a similar tendency irrespective of sex.

The higher expression of Cav1.2α at the base vs apex is observed in women and in rabbits and so are increases in NCX1. One might speculate that the higher influx of Ca^2+^ occurring through I_Ca,L_ is somewhat balanced by the higher Ca^2+^ efflux through I_NCX_. While there are sex differences in Ca^2+^ handling across the plasma membrane, little is known regarding sex differences in Ca^2+^ transport across the sarcoplasmic reticulum. In women, SERCA2a was more abundant at the base compared to the apex, and this large spatial gradient was not observed in men or postmenopausal women; in contrast, there were no spatial gradients of RyR2 in all three groups. The gradient of SERCA2a seen in women was not observed in female but was striking in male rabbit hearts (Fig. 2). Further studies will be needed to determine the functional consequences of these regional sex differences in SERCA2a in terms of the amplitude and rate of SR Ca^2+^ uptake and whether these differences are modulated by sex hormones.

Limitations of availability and viability in culture of healthy human ventricular myocytes precluded studying the estrogen response of isolated myocytes from the base and apex of normal human hearts under voltage clamp. Instead, we tested human iPS-CMs which are known to retain many features of adult ventricular myocytes [34]. As shown here, estrogen-treated female human iPS-CMs exhibited a genomic upregulation of Cav1.2α1 and NCX1 akin to cells from the base of the heart, even though heterogeneity of iPS-CMs may obscure larger changes associated with regional differences of the heart. At physiological membrane potentials (+ 10 to − 90 mV), I_NCX_ was considerably greater in male than in female human iPS-CMs (for instance, I_NCX_ = 0.19 pA/pF in females and − 1.2 mV in males; at − 80 mV). Incubation with estrogen tended to reduce I_NCX_ in male and significantly increased I_NCX_ in female iPS-CMs. The endogenously greater I_NCX_ in males could be due to a number of factors involving iPS-CM maturation and intracellular ionic concentrations.

Unfortunately, sex hormone levels of the organ donors are unknown, and we can only surmise that the donors had normal physiological levels. Nevertheless, the myocardium of young adult females exhibited regional differences in the expression of Cav1.2α1 and NCX1, a profile that was absent in men and in postmenopausal women.

The lack of regional differences in ion channel and calcium-handling protein expression in postmenopausal hearts (reduced estrogen levels) also serves as corroborating evidence that the heterogeneities observed in young adult women are due to genomic regulation by estrogen, similar to findings in female rabbits.

There is an extensive literature focused on the effects of sex steroids on K^+^ channels and QT intervals and related to sex differences in arrhythmia risk. But most studies have rarely paid attention to regional (base-apex) differences in ion channel distribution, calcium-handling mechanisms, or genomic regulation [41]. Studies on repolarizing K^+^ currents explain differences in action potential duration but not the initiation of EADs which trigger TdP. Indeed, the formation of EADs has been shown to result from cellular elevation of Ca^2+^ that activates I_NCX_ (forward mode) and elicits the reactivation of I_Ca,L_. Our study is the first to demonstrate that base-apex differences on the epicardium of the human heart are sex-dependent and occur only in adult women and that these differences trump transmural heterogeneities, which, if they occur, do not seem to be sex-related. Therefore, the higher incidence of drug-induced TdP in women and in female animals could rather be explained by a base-apex heterogeneity of ion channel and calcium-handling protein expression on the epicardium.

## Conclusions

It has long been assumed that sex differences in arrhythmia risk in cases of repolarization delay (e.g., bradycardia and long QT) was due to a “reduced repolarization reserve,” (e.g., a suppression of repolarizing K^+^ currents) in female hearts. However, optical mapping revealed that in rabbit hearts, these arrhythmias were initiated by abnormalities in Ca^2+^ handling. Further studies showed that estrogen imparted these sex differences in adult females through a genomic upregulation of the L-type Ca^2+^ channel and the Na^+^-Ca^2+^ exchanger. These findings altered the mechanisms of action of class III anti-arrhythmic agents and shifted to Ca^2+^ overload as a target to suppress torsade de pointes. The central goal of this study is to examine the human relevance of findings obtained in rabbit hearts. In principle, equivalent experiments would require tissue and cell isolation from different regions of healthy human hearts to measure the genomic effects of estrogen. Instead, we used postmortem myocardium from healthy hearts to analyze sex differences in ion channel expression and human cardiomyocytes derived from induced pluripotent stem cells to measure the functional effects of estrogen on Ca^2+^ currents. The data shows that there is a close correspondence between rabbit and human myocyte responses to estrogen at very low estrogen concentrations. These genomic effects of estrogen at 1 nM trump the acute effects of estrogen on ionic currents observed at micromolar concentrations. Hence, the higher risk of women to torsade de pointes may be caused by a greater Ca^2+^ overload in conditions of repolarization delays. Estrogen binding to estrogen receptor α may act as a transcription factor to upregulate channel expression and promote Ca^2+^ overload. From a public health point of view, the study suggests that Ca^2+^-handling proteins may be important targets to suppress torsade de pointes.

## Abbreviations

Cav1.2α: Primary subunit of the L-type Ca^2+^ channel protein
CMs: Cardiomyocytes
E2: Estrogen or 17-β-estradiol
I_Ca,L_: L-type calcium current
I_NCX_: Sodium-calcium exchange current
iPS-CM: CM derived from induced pluripotent stem cells
LQTS2: Long QT syndrome type 2
NCX1: Sodium-calcium exchange protein
TdP: Torsade de pointes
